# Chemical Genetics Reveals Bacterial and Host Cell Functions Critical for Type IV Effector Translocation by *Legionella pneumophila*

**DOI:** 10.1371/journal.ppat.1000501

**Published:** 2009-07-03

**Authors:** Xavier Charpentier, Joëlle E. Gabay, Moraima Reyes, Jing W. Zhu, Arthur Weiss, Howard A. Shuman

**Affiliations:** 1 Department of Microbiology, Columbia University Medical Center, New York, New York, United States of America; 2 Departments of Medicine and of Microbiology and Immunology, Howard Hughes Medical Institute, Rosalind Russell Medical Research Center for Arthritis, University of California, San Francisco, California, United States of America; Yale University School of Medicine, United States of America

## Abstract

Delivery of effector proteins is a process widely used by bacterial pathogens to subvert host cell functions and cause disease. Effector delivery is achieved by elaborate injection devices and can often be triggered by environmental stimuli. However, effector export by the *L. pneumophila* Icm/Dot Type IVB secretion system cannot be detected until the bacterium encounters a target host cell. We used chemical genetics, a perturbation strategy that utilizes small molecule inhibitors, to determine the mechanisms critical for *L. pneumophila* Icm/Dot activity. From a collection of more than 2,500 annotated molecules we identified specific inhibitors of effector translocation. We found that *L. pneumophila* effector translocation in macrophages requires host cell factors known to be involved in phagocytosis such as phosphoinositide 3-kinases, actin and tubulin. Moreover, we found that *L. pneumophila* phagocytosis and effector translocation also specifically require the receptor protein tyrosine phosphate phosphatases CD45 and CD148. We further show that phagocytosis is required to trigger effector delivery unless intimate contact between the bacteria and the host is artificially generated. In addition, real-time analysis of effector translocation suggests that effector export is rate-limited by phagocytosis. We propose a model in which *L. pneumophila* utilizes phagocytosis to initiate an intimate contact event required for the translocation of pre-synthesized effector molecules. We discuss the need for host cell participation in the initial step of the infection and its implications in the *L. pneumophila* lifestyle. Chemical genetic screening provides a novel approach to probe the host cell functions and factors involved in host–pathogen interactions.

## Author Summary

Many bacterial pathogens subvert the cellular functions of their host by translocating effector proteins into specific cells. *L. pneumophila* primarily targets the alveolar macrophage in its human host or the unicellular protozoa in its natural environment. The bacterium uses a Type IVB secretion system called the Icm/Dot system to translocate its effectors. In contrast to other injection devices, effector secretion by the Icm/Dot system cannot be triggered without the involvement of a target cell. We hypothesize that activity of the Icm/Dot system responds to some signaling or functional activation by the target cell. To identify the host cell function required for activity of the Icm/Dot system we used a small molecule-mediated perturbation strategy called chemical genetics. We screened more than 2,500 annotated small molecules to identify inhibitors of *L. pneumophila* effector translocation in the macrophage. Many of these molecules inhibited known host cell factors involved in phagocytosis. We also identified host cell factors specifically required for *L. pneumophila* phagocytosis. We further show that phagocytosis of *L. pneumophila* by the macrophage is required to trigger effector translocation by the Icm/Dot system. Our data indicate that participation of the target cell is required to generate an intimate contact that stimulates effector translocation by the Icm/Dot system. The host cell participation in the effector translocation process has implications in the environmental lifestyle of *L. pneumophila*. We propose that relying on an active host cell process to stimulate translocation provides *L. pneumophila* with a test for the fitness of the potential host cell. This could prevent the unwanted delivery of *L. pneumophila* effectors into non-productive hosts.

## Introduction

*Legionella pneumophila* is the causative agent of the acute pneumonia known as Legionnaires' disease [1],[2]. Upon inhalation, *Legionella pneumophila* infects and replicates in alveolar macrophages, leading to inflammation and development of the disease. Within host cells, *L. pneumophila* avoids phagosome-lysosome fusion and manipulates host cell processes to create a specialized phagosome that does not acidify and is suitable for intracellular replication [3]–[5]. The Icm/Dot Type IVB secretion system is required for avoiding phagosome-lysosome fusion and for intracellular multiplication [6],[7]. The Icm/Dot system mediates translocation of multiple effector proteins that are responsible for transforming the nascent Legionella phagosome into a replicative compartment, called the Legionella-containing vacuole (LCV) [8]. After several hours, the LCV acquires characteristics of the endoplasmic reticulum (ER) by intercepting small vesicles that traffic between the golgi compartment and the ER [9]. Although most effector proteins have uncharacterized functions, some have been studied in detail and target multiple host cell processes important for the intracellular survival of *L. pneumophila* [8],[10]. For example, LepA and LepB mediate non-lytic release of *L. pneumophila* from protozoan hosts [11]. RalF, DrrA/SidM, LepB and LidA interfere with regulators of ER to Golgi trafficking [12]–[14]. While many other effectors can also interfere with vesicular trafficking by unknown mechanisms [15]–[17], additional effectors target the host innate immune response [18], phosphoinositide metabolism [19] or ubiquitination [20]. The early requirement of a functional Icm/Dot system suggests that effectors must be rapidly translocated upon encounter of the host cell in order to alter trafficking of the newly-formed phagosome and prevent its fusion with the lysosome [21],[22].

Little is known about the processes or signaling events that trigger translocation of bacterial effectors to host cells. For many bacterial pathogens that use a type III secretion system for effector translocation, active release of the effector molecules in the culture supernatants can be triggered in the absence of the host cell [23]. In contrast, for pathogens with type IV secretion system such as *H. pylori*, *Bartonella spp*, *C. burnetti* and *L. pneumophila*, secretion of effectors has not been detected [24] and it is unclear whether or not these pathogens can secrete as well as translocate effectors. Even though *L. pneumophila* translocates a large repertoire of effector proteins during the course of the infection, none of these effectors are released until it encounters a target host cell [25]. This suggests that functional activation of the Icm/Dot system requires sensing of an appropriate host cell by *L. pneumophila*. Effector translocation might be triggered passively by stimuli provided by the host cell. An alternate view is that effector translocation requires the active participation of the host cell. We sought to gain information about the bacterial and host cell factors required for triggering effector translocation by conducting a perturbation study of effector translocation using libraries of small organic molecules with known targets, an approach called chemical genetics [26],[27]. In this approach, thousands of small molecules with known protein targets are screened to identify which proteins are involved in regulating a particular process. This requires annotated libraries of known inhibitors as well as a high-throughput screening assay for the investigated process. We used a β-lactamase reporter system to monitor type IVB effector translocation and adapted it to high-throughput screening. Screening a collection of three commercial libraries consisting of over 2500 bioactive molecules identified a relatively small number of molecules that inhibit translocation. Many of these inhibitors have eukaryotic targets, and some of these inhibit various stages of actin cytoskeleton assembly required for phagocytosis. We show that the triggering of effector translocation is dependent upon phagocytosis and requires the active participation of the host cell. Also, to gain more insight into the parameters that control effector translocation we developed a kinetic translocation assay, which enables detection of effector translocation in real time. We show that host cell contact is a limiting factor for Icm/Dot-dependent effector translocation and that artificially induced intimate contact using the antibody-Fc receptor interaction can increase effector translocation rates by more than 10-fold. Our results reconcile previously conflicting results and provide evidence that phagocytosis-mediated intimate contact of *L. pneumophila* with host cells is critical for efficient effector translocation. We propose a model in which *L. pneumophila* relies on the host cell-dependent phagocytosis to create the intimate binding required to trigger effector translocation.

## Results

### Measurement of *L. pneumophila* effector protein translocation with the β-lactamase reporter system

**Figure 1:**
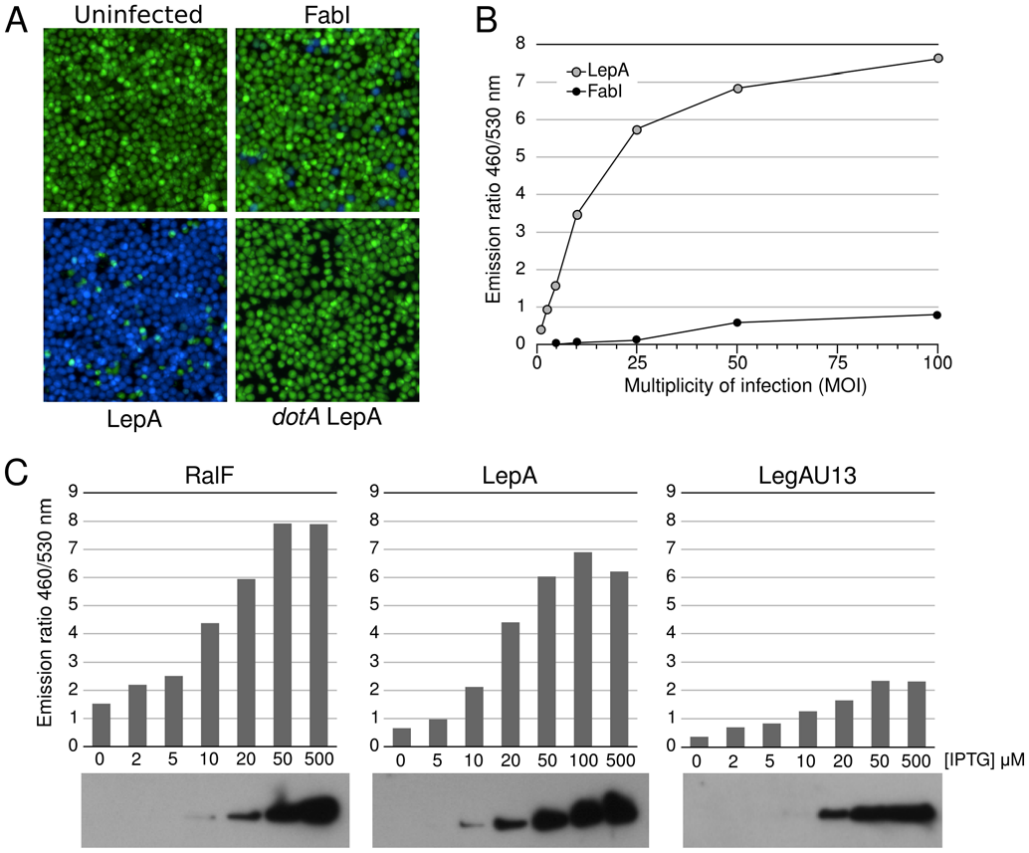
Use of the β-lactamase reporter system to study *L. pneumophila* effector translocation in macrophages. A. Detection of translocation of the previously identified Legionella effector LepA. TEM-LepA or TEM-FabI fusions were produced in *L. pneumophila* and the bacteria were used to infect J774 cells for one hour at 37°C (MOI = 50). Cells were then incubated with the β-lactamase substrate CCF4/AM for two hours at room temperature. Fluorescence images were captured at 460 and 530 nm up 405 nm excitation and merged. B. Measurement of LepA translocation in J774 cells as function of multiplicity of infection. C. Measurement of RalF, LepA and LegAU13 translocation in J774 cells (MOI = 20) as function of TEM fusion protein expression. Bacteria were grown in the presence of varying amount of IPTG to obtain a range of increased TEM fusion protein expression.

The β-lactamase translocation reporter system [28] has been widely used to monitor effector translocation by type III secretion systems in various organisms [29]–[32]. We recently reported the use of this system to detect Icm/Dot-dependent effector translocation by *L. pneumophila* [16]. Host cells are infected with bacteria expressing an effector fused to the C-terminal end of the TEM-1 β-lactamase. The translocated β-lactamase activity in host cells can be quantified using a spectrofluorimeter to measure the concentrations of the cleaved (Emission at 460 nm) and intact (Emission at 530 nm) β-lactamase substrate CCF4. We further validated the reporter system by testing the translocation of the previously reported Icm/Dot effector proteins RalF, LepA, LepB, VipA, VipD, LidA as well as the non-translocated fatty acid biosynthetic enzyme, enoyl-CoA reductase (FabI). As expected, β-lactamase activity was detected in J774 cells infected with bacteria (multiplicity of infection MOI = 50) expressing the various TEM-effector fusions but not in J774 cells infected with the bacteria expressing TEM-FabI (Figure 1A and data not shown). In order to determine the sensitivity and the linearity of the assay we infected J774 cells with increasing numbers of bacteria expressing the translocated TEM-LepA fusion protein. When J774 cells are infected with less than 10 bacteria/cell the system seems to behave linearly but above 25 bacteria/cell the system appears to saturate as increases in MOI do not result in a corresponding increase in the Em460/530 ratio (Figure 1B). As a compromise between sensitivity and linearity, we selected MOI = 20 as the standard MOI in subsequent experiments. We also investigated the effect of protein expression levels on translocation efficiency. *L. pneumophila* expressing TEM-RalF, TEM-LepA or TEM-LegAU13 were grown with varying amounts of IPTG to obtain a range of increased protein expression levels (Figure 1C). In the absence of IPTG no anti-TEM reactive protein could be detected by Western Blot and little or no translocation could be detected. In contrast, translocation of all effectors could be detected when expressed at low levels using 10 µM IPTG. Increasing expression levels resulted in increased translocation up to 50 µM IPTG, thus indicating that effector production is a limiting factor. However, further increases in protein expression levels did not result in increased effector translocation. This plateau in translocation suggests that each effector has a maximal rate of delivery to the host and that when effector protein expression is not limiting, one or more additional unknown factors limit effector translocation. In order to use the β-lactamase translocation reporter assay for chemical genetic screening we miniaturized it to the 384-well format and evaluated its use for high throughput screening (HTS). The Z-factor is a widespread measure of the quality or power of a HTS assay [33]. We found the TEM reporter to be a robust screening assay with a Z factor >0.75 (data not shown), thus making it suitable for high-throughput screening of inhibitors of effector translocation.

### Screening of known bioactive molecules and identification of translocation inhibitors

**Figure 2:**
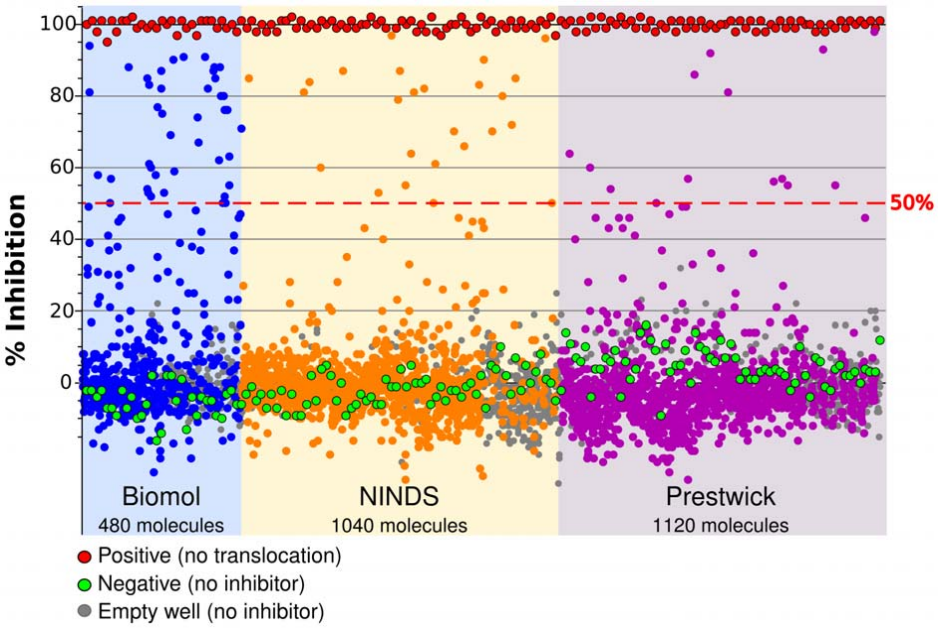
Effect of the three libraries compounds on translocation of the LepA effector. Mean percent inhibition (of two replicates) of translocation for each compound is plotted (blue circles, Biomol compounds; Orange circles, NINDS compounds; Pink circles, Prestwick compounds), along with untreated negative control wells (green circles) and positive control wells lacking *L. pneumophila* cells (red circles). The horizontal bar indicates 50% inhibition of signal and separates active compounds from inactive compounds and negative controls.

In order to identify processes required for effector translocation, we took advantage of three libraries of small molecule inhibitors with a wide variety of known prokaryotic and eukaryotic targets. The Biomol ICCB Known Bioactives Library is a collection of 480 diverse biologically active compounds with defined biological activity and was developed in collaboration with the Harvard Institute of Chemistry and Cell Biology. The NINDS custom collection of 1,040 characterized bioactive compounds was compiled by MicroSource Discovery Systems for the National Institute of Neurological Disorders and Stroke (NINDS). Three quarters of the compounds in the collection are FDA-approved for use in humans. The Prestwick Chemical Library® contains 1,120 off-patent compounds, 90% being marketed drugs. The three libraries together represent 2,640 molecules (see Table S1 for complete list) and cover 46% of FDA-approved active molecules (561 compounds). We screened the three libraries for molecules that inhibited translocation of the LepA effector from Legionella to macrophages (Figure 2). We selected hits showing more than 50% inhibition. In spite of its small size the Biomol library showed the highest hit rate (8.3%) compared to the larger NINDS and Prestwick libraries (hit rates of respectively 1.2% and 2.2%). Because of its small size, its diversity and a high hit rate the Biomol library appears to be most suited library for the identification of inhibitors of a particular biological process.

### Inhibitors of Icm/Dot effector translocation target host cells

**Figure 3:**
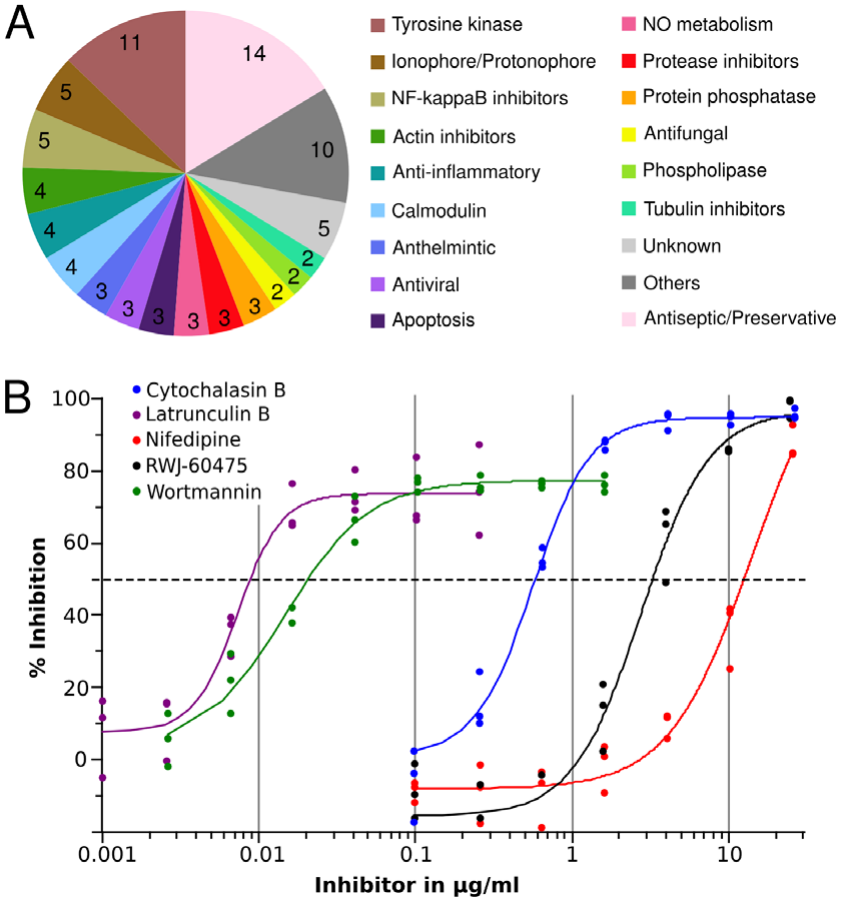
A. Categories or targets of inhibitors identified in initial chemical genetic screen. Number of inhibitors in each class is indicated. B. Dose-response curve of a selected inhibitors on LepA effector translocation. Data from three independent experiments (colored circles) were fitted (colored line) using the four parameter logistic model to determine the IC50.

Initial screening identified 86 translocation inhibitors which are associated with diverse biological categories (Figure 3A and Table S2). We excluded those inhibitors whose documented targets are not well established or predicted to be irrelevant for translocation. These included molecules annotated as antifungal, antiviral or anthelmintic as well as known preservatives (*i.e.*, thimerosal). The remaining 42 inhibitors were retested under non-HTS conditions and one compound was found to be a false positive. Other compounds were found to have been falsely identified as inhibitors because they interfered with the translocation assay itself (fluorescence quencher, autofluorescent molecules, inhibitors of CCF2/AM loading). In order to identify molecules displaying potential off-target effects we determined the IC_50_ of the remaining 25 inhibitors as exemplified in Figure 3. For inhibitors with documented IC_50_ we compared the published values to the determined IC_50_ for the translocation. This led us to exclude three inhibitors (DPI, Cantharidin and MBCQ) which displayed IC_50_ more than one order of magnitude higher than the documented IC_50_. The selected 22 remaining molecules were capable of inhibiting LepA translocation with varying efficiency, ranging from 63 to 100% inhibition (Table 1). All but one of the molecules identified in this study are inhibitors of eukaryotic processes, revealing an unexpected active role for the host cell in the Icm/Dot effector translocation process. A large class of inhibitors target proteins of the cytoskeleton (actin and tubulin) as well as proteins involved in cytoskeleton dynamics (PI3 kinase, N-Wasp). Other molecules target cell surface proteins such as the PDGF receptor or the CD45 tyrosine phosphatase which could be viewed as potential candidate receptors for *L. pneumophila* internalization. Somewhat surprisingly, no antibiotics were identified as inhibitors of translocation in this study. Accordingly, LepA translocation was not inhibited by antibiotics acting as transcription or translation inhibitors, suggesting that pre-synthesized effectors are exported by the Icm/Dot system at early stages of infection.

**Table 1:** List of identified inhibitors of effector translocation.

Inhibitor	Description	Category	µM	% Inhibition	IC50 (µM)
Albendazole	Inhibitor of tubulin polymerization	Cytoskeleton dynamics	50	80	0.9
Cytochalasin B	Binds to and inhibits the association and dissociation of actin filaments	Cytoskeleton dynamics	10	79	1
Fenbendazole	Binds to tubulin and blocks polymerization of tubulin into microtubules	Cytoskeleton dynamics	50	73	0.9
Latrunculin B	Inhibits actin polymerization. 10- to 100-fold more potent than the cytochalasins	Cytoskeleton dynamics	10	86	0.02
LY 294002	A potent and specific inhibitor of phosphatidylinositol 3-kinase	Cytoskeleton dynamics	40	87	9.5
Wiskostatin	Inhibitor of N-WASP, blocks actin filament assembly and prevent the activation of Arp2/3	Cytoskeleton dynamics	25	98	9.4
Wortmannin	Potent and selective inhibitor of phosphatidylinositol 3-kinase	Cytoskeleton dynamics	2	84	0.03
Calcimycin	Highly selective calcium ionophore	Ionophore or Protonophore	20	81	3.5
FCCP	A potent reversible inhibitor of oxidative phosphorylation	Ionophore or Protonophore	15	97	2.9
Nifedipine	Nifedipine, selectively blocks the voltage-sensitive (L-type) calcium channel	Ionophore or Protonophore	50	62	39.9
Phenoxybenzamine	Irreversibly inhibits calmodulin. Selective α-adrenergic blocker	Calmodulin	50	85	15.7
W-7	Binds to calmodulin, inhibiting Ca2+-calmodulin-regulated enzyme activity	Calmodulin	45	98	17.7
CAPE	Inhibits nuclear translocation of NF-κB	NF-kappaB	50	92	9.4
Parthenolide	Sesquiterpene lactone, inhibits activation of MAP kinase and NF-κB	NF-kappaB	30	96	6.5
PI-110	Serine protease inhibitor. Inhibits granzymes A, B, and H2-4 and cathepsin G5	Serine protease	80	84	25.3
TPCK	Inhibitor of chymotrypsin-like serine proteases	Serine protease	30	93	4.5
AG-126	Inhibits LPS-induced tyrosine phosphorylation of p42MAPK	Kinase or Phosphatase	80	86	34.5
RWJ-60475-(AM)_3_	Cell-permeable inhibitor of the CD45 tyrosine phosphatase	Kinase or Phosphatase	25	89	4.5
Tyrphostin 9	Inhibitor of the PDGFR tyrosine kinase and a potent uncoupler of oxidative phosphorylation	Kinase or Phosphatase	25	101	3.5
Furoxan	A NO donor and potent vasodilator and inhibitor of platelet aggregation	Others	20	92	3.9
HA14-1	Bcl-2 ligand which antagonizes its function and induces apoptosis	Others	10	85	3.9
Mas 7	Activator of G proteins by a mechanism analogous to that of G protein-coupled receptors	Others	12	79	6.7

Inhibition of translocation for each inhibitor at the indicated concentration is averaged from three independent experiments.

Even though most inhibitors have a eukaryotic target, one molecule, FCCP, can also target bacterial cells. FCCP acts as a protonophore on the bacterial membrane thus collapsing the proton motive force (PMF).

### Protonophores inhibit the Icm/Dot system activity

**Figure 4:**
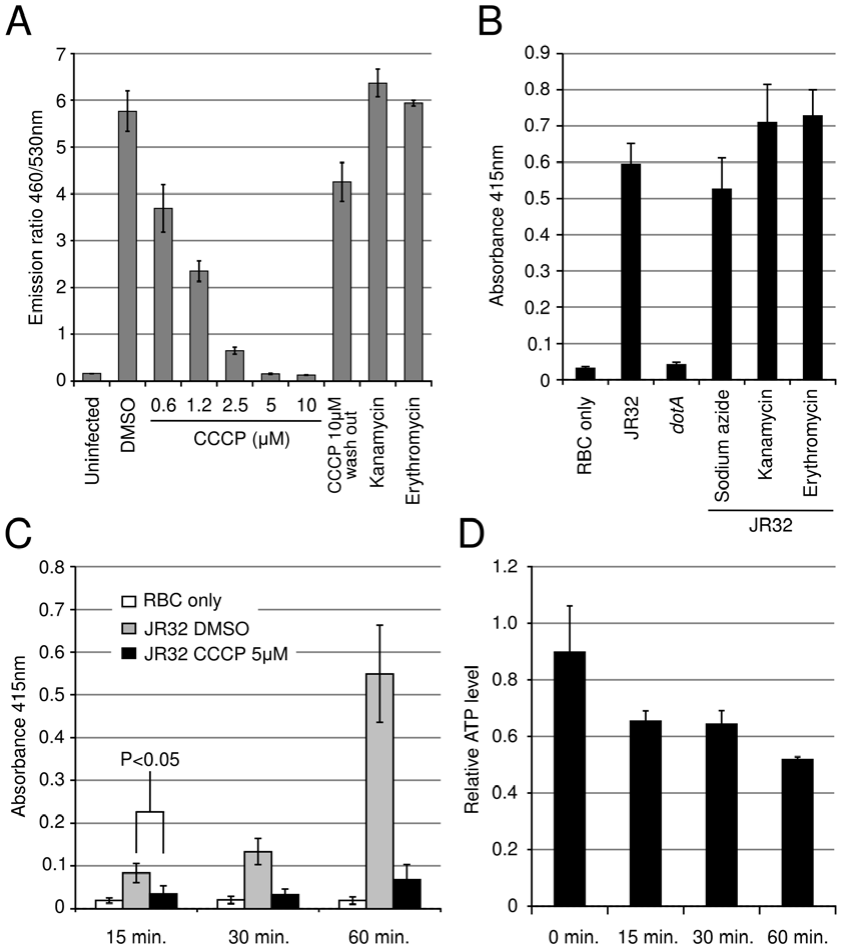
The protonophore CCCP inhibits Icm/Dot-dependent activities. A. Effect of translation inhibitors (kanamycin, erythromycin) and protonophore (CCCP) on translocation of the LepA effector in J774 cells. B. Icm/Dot-dependent red blood cell (RBC) lysis by *L. pneumophila* and effect of translation inhibitors (kanamycin, erythromycin) and respiratory chain poison sodium azide. C. Time course effect of the CCCP protonophore or the DMSO carrier on Icm/Dot-dependent RBC lysis by *L. pneumophila*. D. Effect of the CCCP protonophore on the bacterial ATP levels as a function of exposure time. In all panels error bars represent standard deviation from three independent experiments.

FCCP and CCCP are structurally related protonophores which act as proton carriers discharging both the electric potential (Δψ) and concentration (ΔpH) components of the PMF. CCCP has been shown to inhibit type III secretion [34] and recent findings demonstrate that the flagellar type III secretion apparatus functions as a proton-driven protein exporter [35],[36]. CCCP completely inhibits LepA translocation at concentration as low as 5 µM (Figure 4A). Even when *L. pneumophila* was pretreated with 10 µM CCCP for 30 minutes, translocation was largely restored following washout of CCCP (Figure 4A) showing reversibility of the inhibition. *L. pneumophila* displays pore forming activity on macrophages and red blood cells that is mediated by the Icm/Dot system [37]. Thus, the activity of the Icm/Dot system can also be assayed by red blood cell lysis. RBC lysis is abolished in a *dotA* mutant but, as effector translocation, it is not inhibited by sodium azide or antibiotics acting as translation inhibitors (Figure 4B). RBC lysis is detectable as early as 15 minutes after *L. pneumophila* contacts the RBC (Figure 4C). In contrast to sodium azide and antibiotics, CCCP inhibits RBC lysis very efficiently and rapidly. Since CCCP collapses the proton gradient, it is expected to impact ATP synthesis by the ATP synthase. CCCP has a moderate impact on the pre-existing ATP pool, reducing it by about 30% after CCCP addition (Figure 4D). The energy requirement for activity of the Icm/Dot system and effector translocation is poorly understood. Since the IcmO/DotB component of the Icm/Dot system shows ATPase activity it is expected that Icm/Dot activity is ATP-dependent. Inhibition by protonophore also suggest that effector export by the Icm/Dot system is at least partially dependent on the proton motive force.

**Figure 5:**
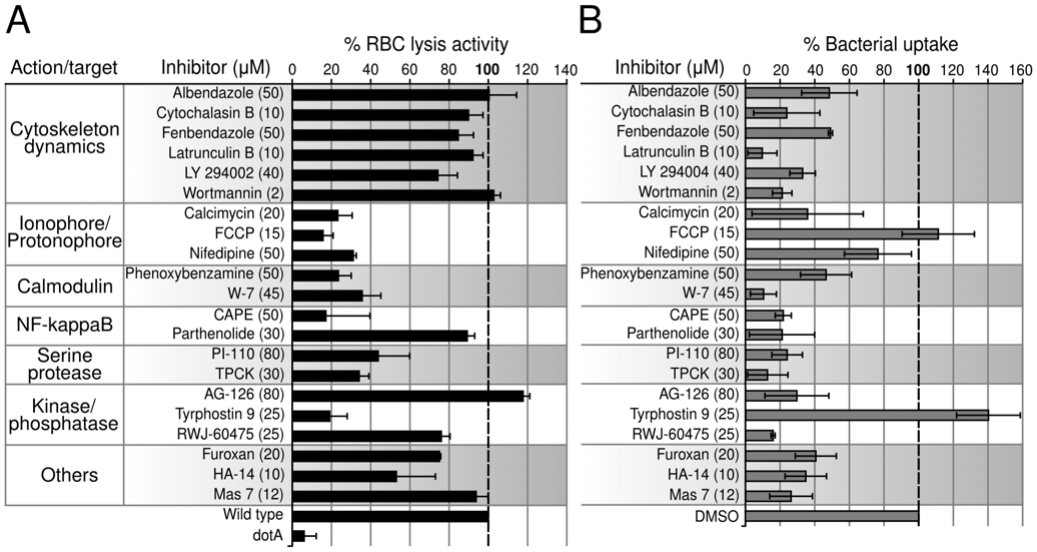
Identified translocation inhibitors inhibit RBC lysis or phagocytosis. A. Effect of the identified translocation inhibitors on Icm/Dot-dependent RBC lysis by *L. pneumophila*. B. Effect of the identified translocation inhibitors on uptake of heat-killed *E. coli* by J774 cells. In both panels the data are reported as the percentage of untreated sample. Error bars represent standard deviation from three independent experiments.

Based on these results, other molecules with protonophore or uncoupling activity are expected to inhibit the Icm/Dot system and effector translocation. We investigated the identified inhibitors of LepA translocation for potential direct inhibition of the Icm/Dot system using the RBC lysis assay (Figure 5A). FCCP showed strong inhibition of RBC lysis as did the calcium ionophore Calcymicin and the calcium channel blocker Nifedipine. Phenoxybenzamine, PI-110, TPCK, W-7 and Tyrphostin 9 activity also strongly reduced RBC lysis activity. Consistent with these findings, the PDGF receptor inhibitor Tyrphostin 9 has documented uncoupling activity. Interestingly, the NF-kappaB (NF-κB) inhibitor CAPE has been previously used to show the requirement of NF-κB for successful *L. pneumophila* infection and intracellular replication [38],[39]. This initially suggested a role of NF-κB in effector translocation during initial interaction with the macrophage. However we found that CAPE also directly inhibits Icm/Dot-mediated RBC lysis (Figure 5A).

### A large class of inhibitors target cytoskeleton dynamics and inhibits phagocytosis

A large number of inhibitors that had no direct effect on Icm/Dot activity are molecules targeting the host cytoskeleton (Tubulin inhibitors, actin inhibitors, PI3K inhibitors). These molecules are likely to alter phagocytosis, a fundamental function of the macrophage. We then investigated the effect of all effector translocation inhibitors on phagocytosis of bacterial particles by using differential, trypan blue-mediated, quenching of fluorescein-labeled *E. coli* [40],[41] (Figure 5B). With the exception of FCCP, Nifedipine and Tyrphostin 9, all the other molecules showed significant inhibition of bacterial uptake (Figure 5B). Inhibition of bacterial uptake was expected for the actin polymerization inhibitors (Latrunculin B, Cytochalasin B) and for the PI3 kinase inhibitors (LY294002, Wortmannin). To a lesser extent, microtubules also play a role in the phagocytosis process [42] where they would be required for activity of PI3K at the site of phagocytosis [43] and may explain the inhibitory activity of Fenbendazole and Albendazole. In contrast, inhibition of bacterial uptake by other molecules such as the inhibitors of NF-κB, CD45 and the serine protease inhibitor or the NO donor Furoxan had not been previously reported. This may be due to a true involvement of the inhibitors targets in the phagocytosis process or to some undocumented off-target effect on phagocytic cells (see discussion). Regardless of the mechanisms of inhibition, the results show that inhibitors of Icm/Dot translocation that do not inhibit Icm/Dot activity itself are enriched in inhibitors of bacterial uptake by the macrophage. This pinpoints a critical role of phagocytosis in Icm/Dot-mediated translocation of LepA by *L. pneumophila*.

### Inhibition of non-opsonic phagocytosis prevents effector translocation

**Figure 6:**
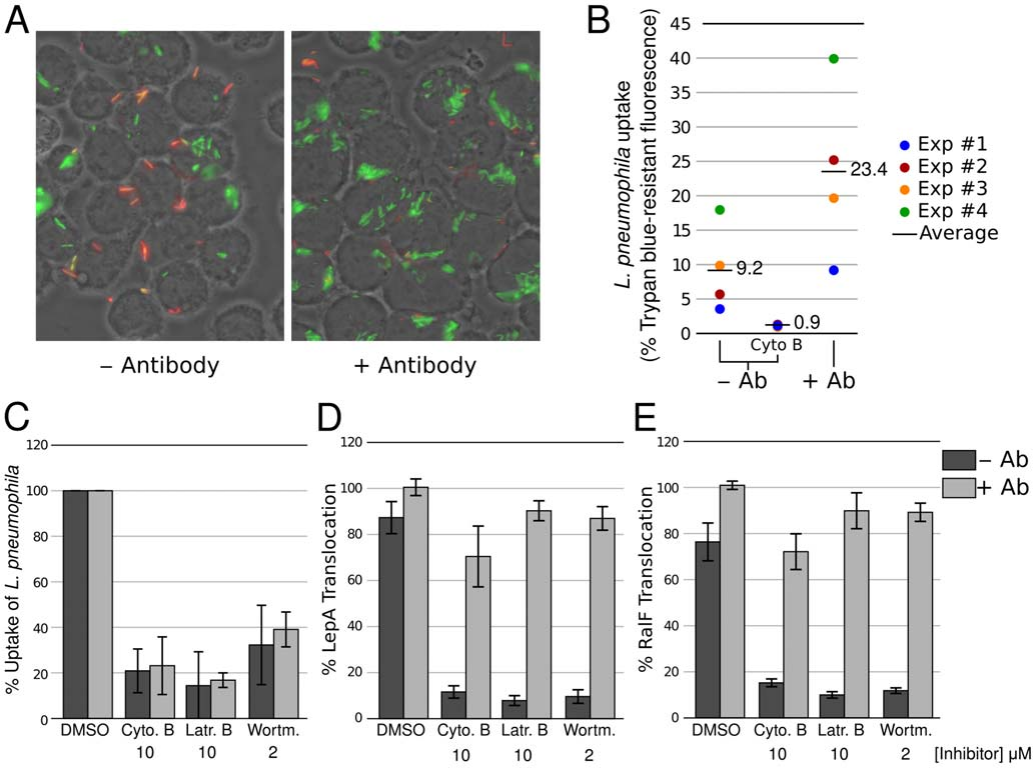
Antibody opsonization of *L. pneumophila* restores effector translocation in the absence of phagocytosis. Effect of antibody-mediated opsonization of *L. pneumophila* on uptake of *L. pneumophila* in J774 macrophages (A and B) and on effector translocation (C). A. Fluorescence microscopy of GFP-expressing *L. pneumophila* (JR32) infecting J774 cells in the absence or presence of anti-*L. pneumophila* antibodies. Extracellular bacteria were labeled with a rhodamine conjugated antibody. B. Uptake of fluorescein-labeled JR32 by J774 cells in the absence or presence of anti-*L. pneumophila* antibodies. Fluorescence signal of extracellular bacteria was quenched with trypan blue. Uptake is reported as the percentage of fluorescence signal that can not be quenched by trypan blue (i.e. the % of intracellular fluorescence signal). C. Effect of phagocytosis inhibitors or the DMSO carrier on *L. pneumophila* uptake by J774 cells in the absence or presence of anti-*L. pneumophila* antibodies. Uptake is reported as the percentage of untreated sample. D and E. Effect of phagocytosis inhibitors or the DMSO carrier on TEM-LepA (D) and TEM-RalF (D) effector translocation in J774 cells in the absence or presence of anti-*L. pneumophila* antibodies. Translocation is reported as the percentage of untreated sample. In all panels error bars represent standard deviation from three independent experiments.

Phagocytosis of *L. pneumophila* by pulmonary macrophages is thought to naturally occur via non-opsonic events and under these conditions *L. pneumophila* has been found to enter monocytes by coiling phagocytosis [44]. Interaction of *L. pneumophila* can be stimulated by Legionella specific antibodies [45], a situation that results in conventional FcR-mediated phagocytosis, but that does not reflect the biologically relevant interaction with host cells. We tested the effect of *L. pneumophila*-specific antibody on *L. pneumophila* phagocytosis by J774 cells using differential immuno-fluorescence microscopy [46]. Internalized GFP-expressing *L. pneumophila* appear green whereas non-internalized *L. pneumophila* which are accessible to rhodamine-conjugated antibody against Legionella appear red/orange. Opsonization of *L. pneumophila* resulted in a higher number of internalized bacteria (Figure 6A). We also used differential, trypan blue-mediated, quenching of fluorescein-labeled *L. pneumophila* to measure the effect of antibody-opsonization on *L. pneumophila* internalization. In four independent experiments, we found that although the level of phagocytosis by J774 cells was variable, antibody-opsonization of *L. pneumophila* always increased phagocytosis by two- to three-fold in all cases (Figure 6B). This result is consistent with previous work reporting that monocyte monolayers infected with *L. pneumophila* contain three times as many bacteria in the presence of antibody [47].

To investigate the role of phagocytosis on effector translocation, we analyzed the effect of inhibitors of actin polymerization and phosphoinositide 3-kinases (PI3K) under non-opsonized and artificial, antibody-opsonized conditions. The two inhibitors of actin polymerization, Cytochalasin B and Latrunculin B dramatically reduce uptake of *L. pneumophila* by J774 cells, either in the presence or absence of antibody (Figure 6C). Phagocytosis of *L. pneumophila* has been initially reported to be insensitive to PI3K inhibitors in human monocytes [48]. A similar result was obtained in Dictyostelium by comparing phagocytosis of *L. pneumophila* by wild-type Dictyostelium and a mutant lacking the two known PI3K [19]. Both strains showed similar levels of phagocytosis of wild-type strain JR32. The requirement for PI3K for phagocytosis by mammalian cells reported here however appears to be different than in Dictyostelium. A recent report shows that phagocytosis of *L. pneumophila* by the murine monocyte cell line J774 is mediated by PI3K and is therefore sensitive to PI3K inhibitors [49]. Consistent with the later study, we found that phagocytosis of *L. pneumophila* can be inhibited by PI3K inhibitors LY294002 at 40 µM (not shown) and wortmannin at 2 µM (Figure 6C). Importantly, all the tested inhibitors inhibited *L. pneumophila* phagocytosis even in the presence of antibodies. We then analyzed the impact of the phagocytosis inhibitors on translocation of LepA and RalF under non-opsonized or antibody-opsonized conditions (Figure 6D and 6E). All tested phagocytosis inhibitors have a dramatic impact on translocation of LepA and RalF under non-opsonized conditions. The same inhibitors also inhibit translocation of LepA in the presence of human complement (data not shown). In contrast, translocation of both LepA and RalF is resistant to phagocytosis inhibitors under antibody-opsonized conditions (Figure 6D and 6E). In conclusion, translocation of Legionella effectors requires phagocytosis under standard, non-opsonized or complement-opsonized (not shown) conditions. However, the requirement for phagocytosis can be bypassed by the presence of anti-Legionella antibody, suggesting that effector translocation can occur in the absence of phagocytosis by promoting *L. pneumophila* binding to macrophages via antibody-Fc receptors interaction.

### The receptor protein tyrosine phosphate phosphatases CD45 and CD148 are specifically required for efficient *L. pneumophila* phagocytosis and effector translocation

**Figure 7:**
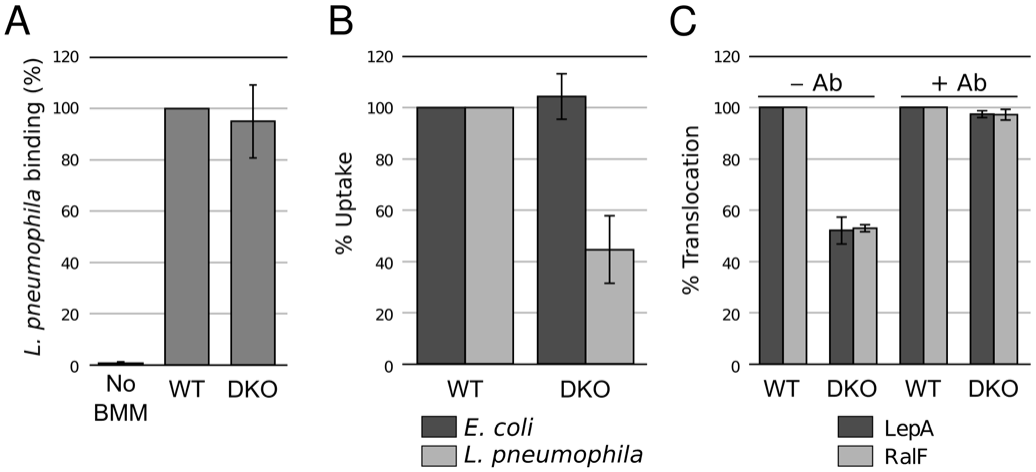
CD45/CD148-deficient bone marrow-derived macrophages are defective for *L. pneumophila* phagocytosis. A. Binding of *L. pneumophila* to WT and CD45/CD148-deficient bone-marrow derived macrophages (BMM). Errors bars represent standard deviation from six replicates of two independent experiments. B. Phagocytosis of *E. coli* and *L. pneumophila* by WT and CD45/CD148-deficient BMM. Error bars represent standard deviation from three experiments. C. Translocation of the TEM-LepA and TEM-RalF effectors in WT and CD45/CD148-deficient BMM (MOI = 10). Errors bars represent standard deviation from six replicates of two experiments.

As a result of chemical screening we found that the small molecule RWJ-60475 inhibits *L. pneumophila* effector translocation. This suggested that its known target, the receptor protein tyrosine phosphate phosphatase CD45, is required for *L. pneumophila* phagocytosis. To investigate the role of CD45 in *L. pneumophila* uptake and effector translocation we used siRNA to knock-down expression of CD45 in THP-1. Although siRNA treatment was effective in significantly lowering expression of CD45 to non-detectable levels, *L. pneumophila* effector translocation remained unaffected (Figure S1). However, it has recently been reported that CD45 and another receptor protein tyrosine phosphate phosphatase, CD148 functioned redundantly to regulate B cell and macrophage immunoreceptor signaling [50]. We used bone-marrow derived macrophages (BMM) from *Ptprj^TM−^* ^/*TM−*^, *Ptprc* ^−/−^ mice which do not express CD45 and CD148 to determine if these tyrosine phosphate phosphatases also functioned redundantly to mediate *L. pneumophila* binding and uptake. *L. pneumophila* bound similarly to wild-type and DKO BMM (Figure 7A) suggesting that CD45 and CD148 do not constitute receptors for *L. pneumophila* binding to macrophages. In contrast, uptake of *L. pneumophila* by CD45/CD148-deficient BMM was significantly reduced (Figure 7B) while the CD45/CD148 double knockout (DKO) BMM showed unaltered ability to phagocytose *E. coli* bacteria (Figure 7B). Translocation of the two effectors RalF and LepA in the DKO BMM was reduced to the same extent as uptake (Figure 7C). Antibody opsonization restored effector translocation in the CD45/CD148-deficient BMMs. These results reveal the important role of tyrosine phosphate phosphatases for *L. pneumophila* entry into macrophages and provide strong genetic evidence that *L. pneumophila* phagocytosis is a critical event for delivery of Icm/Dot effector proteins. It should be noted that inhibition of bacterial uptake by RWJ-60475 is greater than the inhibition displayed by the CD45/CD148-deficient BMMs and suggests that RWJ-60475 acts on the phosphatase activities of CD148 as well as on other as yet unrecognized tyrosine phosphatases.

### Triggering of translocation in the presence of antibody does not require Fc receptor signaling

**Figure 8:**
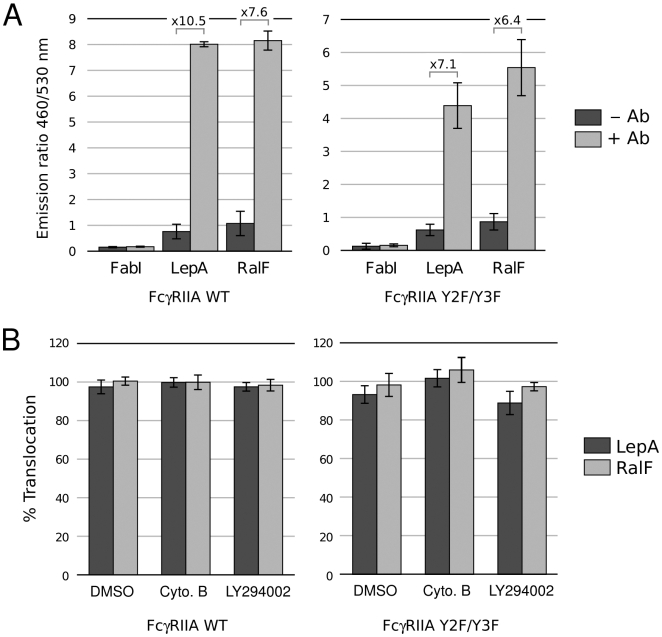
Effector translocation in the presence of antibody does not require immunoreceptor signaling. A. Antibody-stimulated translocation of the LepA and RalF effector in CHO cells expressing the wild-type or signaling-deficient (Y2F/Y3F) FcγRIIA. B. Effect of phagocytosis inhibitors or the DMSO carrier on antibody-stimulated LepA and RalF translocation in CHO cells expressing the wild-type or signaling-deficient (Y2F/Y3F) FcγRIIA. Error bars represent standard deviation from three independent experiments.

The fact that phagocytosis is dispensable for translocation when bacteria contact macrophages through the antibody-FcR interaction suggests that either the tight binding of the bacteria to the cells or signaling events due to clustering of the high affinity Fc receptors, are promoting effector translocation. Binding of antibody-coated particles to FcR induces clustering of the receptors and initiates a signal transduction cascade leading to phagocytosis [51],[52]. Clustering is then followed by phosphorylation of two specific tyrosines on the FcR ITAM domain (Immunoreceptor Tyrosine-based Activation Motifs) by enzymes of the Src tyrosine-kinase family [53]. The phosphorylated ITAMs then recruit Syk kinase which is required for efficient phosphorylation of phosphatidylinositol 3-kinase and is critical for signal transduction and phagocytosis mediated by FcR [54]. As initial evidence that FcR signaling is not required for antibody-stimulated effector translocation, we found that the Syk kinase inhibitor R406 [55] does not inhibit LepA translocation in J774 cells (data not shown). To further determine if binding of antibody-opsonized bacteria to the FcR is sufficient to trigger effector translocation, we used CHO cells expressing the human FcγRIIA receptor or a signaling deficient mutant of FcγRIIA, Y2F/Y3F in which the two tyrosines of the ITAM domain have been substituted by phenylalanine [56]. Opsonization of Legionella with antibody strongly stimulated translocation of LepA and RalF in cells expressing the wild-type or signaling deficient FcγRIIA receptors (Figure 8A). Whereas the wild-type FcγRIIA is fully functional and capable of promoting phagocytosis, the Y2F/Y3F mutant can bind antibody but does not support phagocytosis [56]. Consistently, translocation of LepA and RalF in CHO cells expressing either the wild type or the signaling-defective receptors is insensitive to phagocytosis inhibitors (Figure 8B). We conclude that the intimate binding of antibody-opsonized *L. pneumophila* to FcR on host cells can substitute for naturally occurring phagocytosis to trigger effector translocation.

### Phagocytosis-dependent effector translocation is rate limited

**Figure 9:**
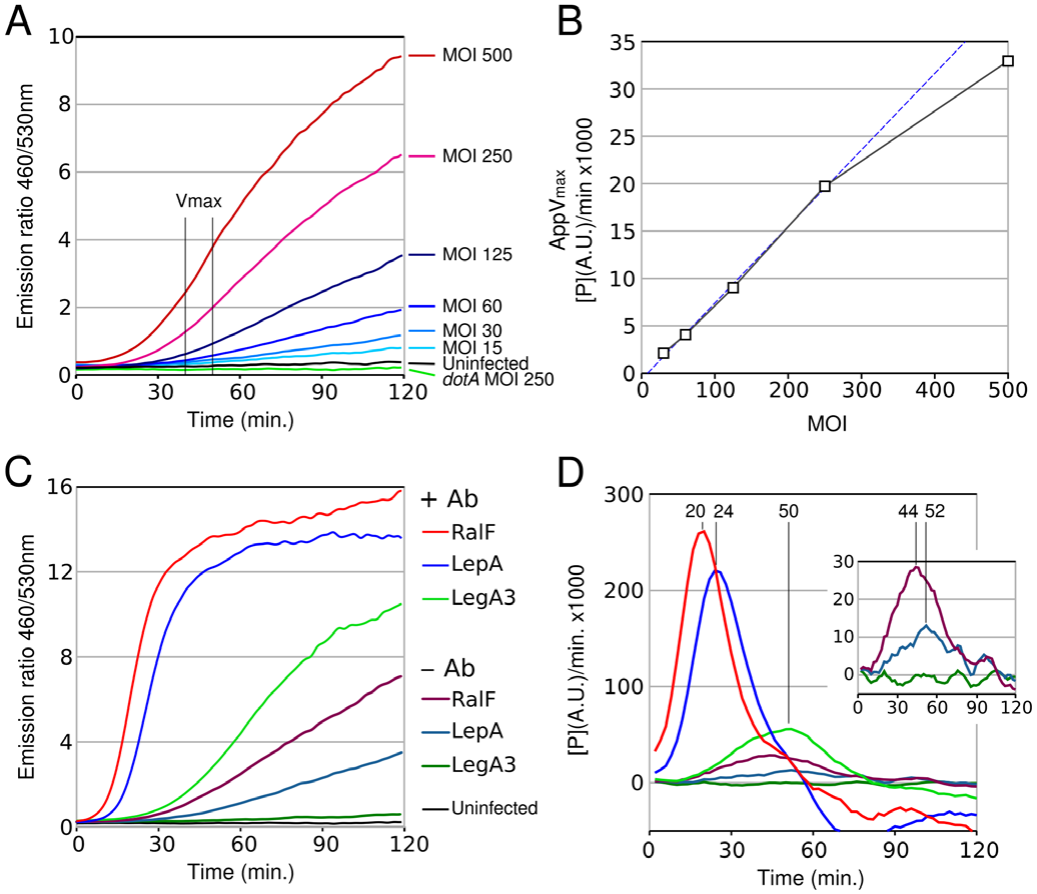
Real time analysis of effector translocation by *L. pneumophila*. A. Real time detection of CCF4 hydrolysis mediated by TEM-LepA translocation in J774 cells from *L. pneumophila* at various multiplicity of infection (MOI). B. Effect of MOI on the apparent maximal rate of product accumulation mediated by translocated TEM-LepA effector. The blue dotted line represents the ideal correlation between MOI and the apparent maximal rate of product accumulation (correlation coefficient of 1). C. Effect of antibody opsonization of the translocation efficiency of TEM-RalF, TEM-LepA and TEM-LegA3. D. Rate of product accumulation mediated by translocated TEM-RalF, TEM-LepA or TEM-LegA3 effectors as function of time in the presence or absence of antibodies. Legend of panel C applies also to panel D. Rates of product accumulation are expressed as arbitrary unit per unit of time (min.). The presented data are from a representative experiment.

In order to better evaluate the effect of antibody-induced bacterial contact on effector translocation we adapted the β-lactamase reporter to study real time translocation of effectors. The β-lactamase translocation assay has recently been used to analyze real-time effector translocation by the EPEC type III secretion system and allowed a more detailed analysis of the parameters that control effector delivery into host cells [57]. In contrast to the standard β-lactamase translocation assay where CCF4 hydrolysis is measured at the end of the infection, the real-time translocation assay is based on live detection of CCF4 substrate hydrolysis as the TEM-effector fusion is being translocated by the infecting bacterial population. J774 cells were pre-loaded with the β-lactamase CCF4 substrate in presence of probenecid which inhibits organic anion transporters [58] and facilitates loading of CCF4 by inhibiting its active efflux from the cells. Cells were then infected with *L. pneumophila* strains expressing β-lactamase-effector fusion proteins and placed into a plate reader at 37°C. Hydrolysis of green CCF4 into its blue product by the translocated TEM-hybrid β-lactamases was then monitored by measuring blue (460 nm) and green fluorescence (530 nm) every 2 minutes and is reported as the 460/530 ratio. Hydrolysis of CCF4 was evident after less than 30 minutes in J774 cells infected with TEM-LepA expressing bacteria at a multiplicity of infection of 125 or higher (Figure 9A). As expected, decreases of the MOI led to decreased accumulation rate of the blue CCF4 hydrolysis product, but was still detectable at a MOI of 15. In contrast, even at high MOI, no translocation was detectable when J774 cells are infected by a *dotA* mutant expressing TEM-LepA. Accumulation of the CCF4 cleavage product (P) per time unit provides the hydrolysis rate of CCF4 by the translocated β-lactamase-effector fusion and an apparent maximum rate (app*V*_max_) can then be determined. Regardless of the MOI, the app*V*_max_ for the LepA effector is typically reached within 40–50 minutes (Figure 9A). As more β-lactamase-effector fusion is being translocated, the app*V*_max_ should be proportional to the MOI and we observed a good correlation of app*V*_max_ with MOI up to an MOI of 250 (Figure 9B). We then analyzed the translocation of RalF, LepA and LegA3 by non-opsonized *L. pneumophila* at a MOI of 125. Translocation of RalF and LepA could easily be monitored in real time (Figure 9C). Translocation of LegA3 was difficult to detect because the rate of LegA3 translocation is slow and is obscured by the leakage of the CCF4 substrate from J774 cells even in the presence of probenecid (Figure 9C). Analysis of the rate of product formation by the translocated TEM-RalF and TEM-LepA hybrid proteins shows a burst at respectively 44 and 52 minutes post-infection (Figure 9D). Under these conditions, phagocytosis is required for effector translocation and the kinetics of effector translocation may be limited by the kinetics of the phagocytosis process. The absence of phagocytosis requirement when contact is achieved by antibody-FcR interaction may remove this rate-limiting kinetic step. As expected, we found that opsonization of *L. pneumophila* by antibody dramatically increased translocation efficiency and increased the rate of product formation by more than 10-fold (Figure 9C and 9D). In addition, the translocation rates for the TEM-RalF and TEM-LepA hybrid proteins reached a maximum at respectively 20 and 24 minutes post-infection which is significantly earlier than in the absence of antibody. In addition, the hierarchy of the translocation rates of the different hybrids is maintained in the presence of antibody (RalF>LepA>LegA3). We conclude that antibody-mediated contact of *L. pneumophila* with macrophages stimulates translocation by providing the direct intimate contact normally obtained through the rate-limiting phagocytosis process.

## Discussion

Delivery of effector proteins capable of interfering with host cell processes is a mechanism widely used by bacterial pathogens to hijack the function of their target cells and cause disease. Effector delivery is achieved by elaborate effector injection devices such as the Type III secretion system [23], the Type IV secretion system [24] and the recently recognized Type VI secretion system [59]. Type III and Type VI secretion of the bacterial effectors respond to environmental stimuli and can be triggered *in vitro* by using various chemicals or media formulations. In contrast, effector secretion by the *L. pneumophila* Type IV secretion system has not yet been detected unless the bacterium encounters a target host cell [25]. We sought to determine the mechanisms involved in the signaling of effector translocation by *L. pneumophila*. Chemical genetics has recently appeared as a successful strategy to generate hypotheses regarding underlying biological mechanisms [60],[61]. We used a combination of three commercially available annotated compound libraries of more than 2,500 molecules to understand the mechanisms involved in triggering effector translocation by *L. pneumophila*. We found that a very limited number of molecules can directly block activity of the Icm/Dot system and that inhibitors of protein synthesis are not effective at inhibiting Icm/Dot-dependent effector translocation and pore forming activity (Figure 4). Thus, activity of the Icm/Dot system does not rely on *de novo* synthesis of structural or effector proteins. This reinforces the notion that the Icm/Dot system is in a “locked and loaded” state before *L. pneumophila* encounters a target cell. Although Icm/Dot-dependent effector translocation and pore forming activity are insensitive to many antibiotics and even to sodium azide, we found that the same Icm/Dot-dependent processes are strongly but reversibly inhibited by the protonophore CCCP. The pH gradient across the bacterial membrane generated by the respiratory chain is used as an energy source to power the flagellar motor and ion antiporters. This proton motive force (PMF) has recently been shown to be required for activity of the Type III secretion system which functions as a proton-driven protein exporter [35],[36]. It is tempting to speculate that the PMF also powers the Icm/Dot system. However, inhibitor-mediated collapse of the PMF also results in a 30% drop of the ATP level which could also explain the loss of Icm/Dot activity. We can think of at least two ways that energy may be required for Icm/Dot- dependent RBC lysis. Energy may be required to assemble a functional TFSS from pre-existing components. Alternatively, PMF may be required to translocate pore-forming protein molecules to the RBC membrane and other target cells. Although PMF would be an immediate and convenient source of energy to assemble or power the Icm/Dot system, the interpretation of experiments using inhibitors of the PMF is challenging and further studies will be required to ascertain the role of PMF in Icm/Dot effector translocation.

Only a very limited number of molecules were capable of acting directly on Icm/Dot activities. In contrast, many more molecules acting on the host cells were effective at inhibiting effector translocation. We found known inhibitors of phagocytosis as well as inhibitors of other processes. With the exception of the inhibitors interfering with RBC lysis, all other inhibitors displayed marked inhibition of phagocytosis of inert bacterial particles in some cases for apparently unclear reasons. For example, inhibition of phagocytosis by the two inhibitors of NF-κB, CAPE and parthenolide was unexpected. Interestingly, another NF-κB inhibitor has been found to negatively affect phagocytosis by murine macrophages [62] suggesting a role of NF-κB regulation of phagocytosis of bacterial particles. In spite of extensive literature on NF-κB we did not find more direct evidence supporting the involvement of NF-κB in the phagocytosis process. Similarly, although RWJ-60475 inhibits bacterial uptake, its known target, the receptor protein protein tyrosine phosphate phosphatase CD45 was not previously known to be required for phagocytosis. In addition, effective siRNA knock-down of CD45 in THP-1 cells failed to provide evidence for a requirement of CD45 for *L. pneumophila* uptake and effector translocation (data not shown). However, we found that macrophages derived from a mutant mouse strain lacking both the CD45 and CD148 protein phosphatases exhibited a defect in *L. pneumophila* phagocytosis similar to that reported for ingestion of RBC by the same type of cells. This suggests that RWJ-60475 may also act on the phosphatase activity of CD148 or other as yet unrecognized phosphatases. Large-scale use of specific inhibitors is thus a powerful tool to generate hypotheses regarding the factors involved in a given process. However, as illustrated above, the possible effect of the inhibitors on additional undocumented targets must be considered.

**Figure 10:**
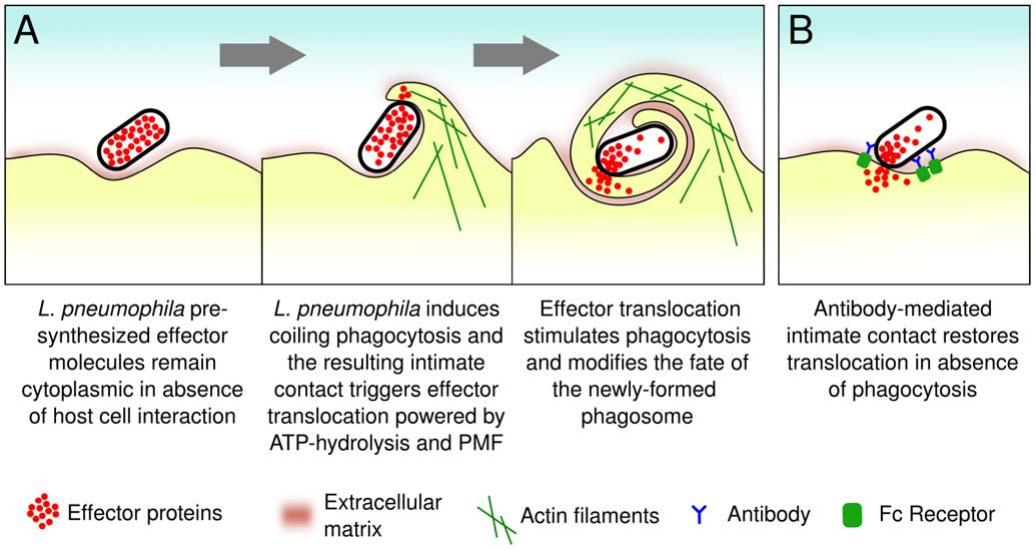
*L. pneumophila* with macrophages under complement-opsonized or non-opsonized conditions (A) and antibody-opsonized conditions (B).

The strong requirement of phagocytosis for *L. pneumophila* effector translocation is remarkable. Other bacterial pathogens inject bacterial effectors without the requirement of phagocytosis and some pathogens like *Yersinia enterocolitica* and enteropathogenic *E. coli* even utilize type III-dependent effector translocation to inhibit phagocytosis [63]–[65]. We found that when macrophages are given non-opsonized Legionella, addition of actin depolymerizing agents that block phagocytosis severely decreased effector translocation. However, when the same cells were given antibody opsonized-Legionella, the actin depolymerizing agents did not inhibit translocation. We can imagine two different explanations for these results. One explanation is that in the presence of antibodies, binding of the bacteria to the macrophages is so efficient that, as seen by Kirby *et al.* [37] even in the presence of actin depolymerizing agents, there is a low level of phagocytosis that corresponds to the level of uptake observed in the absence of antibody and inhibitor. An alternative explanation is that the increased level of Legionella binding to the macrophages in the presence of antibody permits translocation even when the bacteria remain outside the cell in the presence of the inhibitors. We favor the second explanation because we found that opsonized Legionella were able to translocate effectors to CHO cells expressing mutant forms of the FcγRIIA receptor that are defective for signaling and cannot promote phagocytosis. Thus, the signaling cascade induced as a result of antibody-FcR binding is not required to trigger Icm/Dot-mediated effector translocation. Under these conditions, it is highly unlikely that the bacteria are being internalized. This supports the idea that artificially-induced intimate contact can trigger translocation in absence of phagocytosis and that phagocytosis is not required *per se* for translocation. These results provide a resolution for the apparent discrepancy between published work from our lab done with professional phagocytes in the absence of opsonization [25] and work from the Roy lab using CHO(FcγRIIΑ) cells and opsonized Legionella [66]. Real-time analysis of effector translocation showed that phagocytosis-dependent translocation is much slower than the phagocytosis-independent translocation triggered by antibody-FcR intimate binding. Presumably phagocytosis-dependent translocation is slower because the kinetics of phagocytosis adds time to the kinetics of effector translocation. The absence of evidence for protrusion of Icm/Dot system suggest that intimate binding may be needed to overcome the physical barrier established by the extracellular structures present on the surface of the bacterial or cellular membranes. Pulmonary macrophage infection by *L. pneumophila* generally occurs in the absence of pre-immune antibodies. Under *in vivo* conditions it is then likely that *L. pneumophila* relies on host cell signaling to stimulate uptake and achieve intimate contact required for effector translocation. Our data support a model in which *L. pneumophila* relies on the phagocytosis process to generate the intimate contact required for the translocation of pre-synthesized effector molecules (Figure 10). The phagocytosis requirement for effector translocation by *L. pneumophila* has consequences for its environmental lifestyle. Indeed, it is believed that amoebae are natural hosts for *L. pneumophila* providing a critical environment for its replication and survival [67]. *L. pneumophila* may only translocate its effectors when residing in a phagocytic membrane-bound compartment or once phagocytosis has been initiated. The translocated effectors can thereafter alter this compartment so as to create a vacuolar environment permissive for *L. pneumophila* replication. In addition, effector translocation will only be triggered by a potential host cell energetically competent to perform phagocytosis. Therefore relying phagocytosis to trigger translocation ensures that it is occurring only under conditions that could result in a successful infection, and thus, avoid delivery of effector to a host cell that cannot support replication. Interestingly, translocation of a *Vibrio cholerae* type VI secretion effector also requires internalization by host cells [68]. This is also somehow reminiscent of activation of the SPI-2 type III secretion system of Salmonella following internalization by macrophages [69]. Regardless of the secretion system used for translocating effector proteins, bacterial pathogens that interact with phagocytes may have evolved strategies to ensure that translocation occurs only in the phagosomal/endosomal microenvironment.

Chemical genetic screening is a successful strategy to identify host cell functions that are required for effector translocation by *L. pneumophila*. Our study was limited to the initial interaction of the bacteria with host cells but this approach could be applied to the later stages of infection. The commercial availability of annotated libraries of specific inhibitors and FDA-approved drugs will certainly lead to additional studies using chemical genetic screening. Chemical genetic screening should be considered as a additional tool to cellular microbiologists and provides an alternative approach to RNAi-mediated identification of host cell factors required for a bacterial infection.

## Materials and Methods

### Cells and materials

J774A.1 and THP-1 cells were obtained from ATCC and routinely cultivated in RPMI 1640 (Invitrogen) supplemented with 10% fetal bovine serum (FBS). CHO cells expressing the human FcγRIIA or the signaling deficient mutant of Y2F/Y3F [56] were cultivated in Ham's F12 nutrient mixture (Invitrogen) containing 10% FCS and 300 µg/mL G418. Phagocytosis inhibitors were obtained from Biomol (Plymouth Meeting, PA), solubilized in DMSO and used at the following concentrations : Cytochalasin B (10 µM), latrunculin B (10 µM), LY294002 (40 µM), wortmannin (2 µM) and nocodazole (25 µM). *L. pneumophila* strains were grown in liquid media ACES [*N*-(2-acetamido)-2-aminoethanesulfonic acid]-buffered yeast extract (AYE) or on solid media ACES-buffered charcoal yeast extract (CYE) plates. Chloramphenicol and kanamycin were used respectively at 5 µg/mL and 50 µg/mL. Rabbit polyclonal anti-Legionella antibodies were obtained by immunizing rabbit with purified major outer membrane protein (MOMP) [70].

### *L. pneumophila* strains and plasmids

All of the experiment described here were performed with *L. pneumophila* Philadelphia-1 derived strain JR32 [71] or with KS79 [16], a constitutively competent *comR* mutant of JR32. A *dotA* deficient strain of KS79 was constructed by transforming KS79 with genomic DNA extracted from the JR32 *dotA::*Tn903dII*lacZ* strain LELA3118 [71]. Plasmids expressing β-lactamase effector protein fusions were constructed as previously described [16]. Briefly, PCR product of the effector gene was digested with appropriate restriction enzymes and cloned in the KpnI-SmaI-BamHI-XbaI polylinker of pXDC61. Plasmids were transformed into KS79 or KS79 *dotA* by natural transformation [72].

### Analysis of production of TEM fusion proteins

Bacterial lysates were prepared from *L. pneumophila* suspension used for translocation assays (see below). Aliquots were boiled for 5 min. in SDS-PAGE sample buffer and subjected to denaturing polyacrylamide gel electrophoresis. Proteins from SDS-polyacrylamide gels were electrophoretically transferred to nitrocellulose sheets (Schleicher and Schuell) and subsequently stained with Ponceau S (Sigma) to check the loading of the lanes. Sheets were analyzed by Western blotting with monoclonal antibody directed to the TEM-1 β-lactamase (5 µg/mL, QED Bioscience) as a primary antibody and an anti-mouse peroxidase conjugate (20 nG/mL, Pierce) as secondary antibody. Nitrocellulose sheets were revealed with the SuperSignal® West Dura detection system (Pierce) and Biomax films (Kodak).

### Translocation assay in J774 and CHO FcR-expressing cells

Translocation assay in J774 or CHO FcR cells were performed as previously described [16]. Briefly, 24H prior to infection J774 cells grown in RPMI 1640 (Invitrogen) containing 10% FCS were seeded in black clear-bottom 96 well plate at 1×10^5^ cells/well. CHO cells were grown in Ham's F12 nutrient mixture (Invitrogen) containing 10% FCS and seeded in black clear-bottom 96 well plate at 5×10^4^ cells/well. When the infections were performed in presence of antibodies or inhibitors, J774 or CHO cells were pre-incubated for 30 min. prior to infection in complete media containing the anti-Legionella antibodies (1∶200 dilution) and/or inhibitors. *L. pneumophila* strains carrying the various blaM fusions were grown on CYE plates containing choramphenicol (5 µg/mL) and then streaked on CYE plates containing chloramphenicol and 0.5 mM IPTG and grown for 24H to induce expression of the hydrid proteins. Bacteria were resuspended in RPMI 1640 or F12 nutrient mixture to obtain varying MOI (assuming OD = 1 is about 1.4×10^9^ cfu/mL) and each well is infected with 10 µL of the suspension. After centrifugation (900 g, 10 min.) to initiate bacterial-cell contact the plate was shifted to 37°C and incubated for one hour in a CO_2_ incubator. Cell monolayers were loaded with CCF4 by adding 20 µl of 6× CCF4/AM solution (LiveBLAzer™-FRET B/G Loading Kit, Invitrogen) containing 15 mM Probenecid (Sigma). The cells were incubated for 2 hours at room temperature, fluorescence was quantified on an Victor microplate reader (Perkin-Elmer) or Infinite M200 plate reader (TECAN) with excitation at 405 nm (10-nm band-pass), and emission was detected via 460-nm (40-nm band-pass, blue fluorescence) and 530-nm (30-nm band-pass, green fluorescence) filters. Translocation was expressed as the ratio of fluorescence emitted at 460 nm and 530 nm (Emission ratio 460/530).

### HTS-formatted effector translocation assay, screening of bioactive molecules libraries and IC50 determination

Screening of the bioactives libraries was conducted at the National Screening Laboratory for the Regional Centers of Excellence in Biodefense and Emerging Infectious Disease (Boston, MA). On the day preceding the infection J774 cells in were seeded in black clear-bottom 384 well plate (Costar) at 3×10^4^ cells/well in 25 µL of RPMI 1640 containing 10% FCS. *L. pneumophila* carrying the blaM-LepA fusion was grown on CYE plates containing choramphenicol (5 µg/mL) and then streaked on CYE plates containing chloramphenicol and 0.5 mM IPTG and grown for 24H to induce expression of the hybrid protein. Libraries of bioactives were arrayed in 384-well plates at 5 mG/mL in DMSO and 0.1 µL of the molecule solution were transferred per well of the plates containing the J774 cells. Bacteria were then resuspended in RPMI 1640 to OD = 0.21 (3×10^8^ bacteria/mL) each well is infected with 5 µL of the suspension. After centrifugation (900 g, 10 min.) to initiate bacterial-cell contact the plate was shifted to 37°C and incubated for one hour in a CO_2_ incubator. Cell monolayers were loaded with CCF4 by adding 6 µl of 6× CCF4/AM solution (LiveBLAzer™-FRET B/G Loading Kit, Invitrogen) containing 15 mM Probenecid (Sigma). The cells were incubated for 2 hours at room temperature and fluorescence was quantified on an Victor microplate reader (Perkin-Elmer). Screening was carried out in duplicate. IC50 determination were performed similarly using 2.5-fold serial dilution of the considered molecules in 384 well plates. The dose-response data were fitted with the GNU General Public Licensed software QtiPlot using the four parameter logistic model according to the *Assay Guidance Manual Version 5.0* (Eli Lilly and Company and NIH Chemical Genomics Center).

### Red blood cell lysis and measurement of ATP levels

Sheep red blood cell (RBC) were washed in PBS and resuspended in RPMI at at 5×10^7^ RBC/mL and 200 µL were distributed in 96 well plates. Legionella from overnight AYE cultures were resuspended in RPMI at 1×10^9^/mL. The RBC were infected with 50 µL of the bacterial suspension (MOI = 5) by centrifugation at 600 g for 5 minutes and then incubated at 37°C for up to one hour. Where indicated, CCCP was added to the bacterial∶RBC mixture immediately prior to centrifugation. After the incubation, the mixtures of bacteria and RBC were resuspended and then centrifuged again at 600 g for 5 minutes. The absorbance of hemoglobin in the supernatants was then measured at 415 nm. Effect of CCCP (5 µM) on bacterial ATP levels was measured on the same bacterial suspension used in RBC lysis experiment. Before addition of CCCP a 20 uL aliquot was removed and added to 2 µL TCA 10%. After a 5 minute incubation, 20 uL of Tris-Acetate 1 M pH 7.75 were added followed by the addition of 160 µL ice-cold H2O. The same procedure was used immediately after addition of CCCP and after 15, 30 and 60 minutes incubation period at 37°C. ATP levels were quantified using Promega ENLITEN® ATP Assay System Bioluminescence Detection Kit. ATP levels after addition of CCCP were expressed relatively to the untreated sample.

### Phagocytosis assay of *E. coli* and *L. pneumophila* by trypan blue quenching

*E coli* and *L. pneumophila* phagocytosis was measured by trypan blue quenching as previously described [40],[41]. Heat-killed *E. coli* were fluorescent labeled using fluorescein isothiocyanate (FITC) [73]. *L. pneumophila* were fluorescein-labeled with 5,6-Carboxyfluorescein succinimidyl ester (FSE) as previously described [22]. In contrast to fluorescein isothiocyanate (FITC) the FSE reagent does not compromise bacterial viability and allows labeling of live *L. pneumophila* [22]. *L. pneumophila* from 1 mL overnight cultures in AYE were washed three times in 50 mM potassium phosphate (pH 8.0) and resuspended in 1 ml 50 mM potassium phosphate (pH 8.0). FSE was solubilized in DMSO at 10 mG/mL and 10 µL were added to the bacterial suspension. The bacterium-FSE mixtures, in 1.6-ml plastic centrifuge tubes, were periodically inverted throughout a 20-min incubation at room temperature. The reaction was terminated by resuspending the bacteria in M63 salts and washed three times in M63 salts. Live bacteria were finally resuspended at 2×10^8^ cfu/mL in complete RPMI media and fluorescence of the fluorescein-labeled bacteria (10 µL sample) was quantified on an Victor microplate reader (Perkin-Elmer) with excitation at 485 nm (10-nm band-pass filter) and emission at 530-nm (30-nm band-pass filter). 24H prior to infection J774 cells grown in RPMI 1640 (Invitrogen) containing 10% FCS were seeded in black clear-bottom 96 well plate at 1×10^5^ cells/well. Before the infection, media was replaced with fresh complete RPMI 1640 alternatively containing phagocytosis inhibitors and cells were incubated for 30 minutes. Each well is then infected with 10 µL of the bacterial suspension (MOI = 20). After centrifugation (600 g, 10 min.) to initiate bacterial-cell contact the plate was shifted to 37°C and incubated for 30 min. in a CO_2_ incubator. The media was then removed media and replaced with 50 µL/well of a trypan blue solution (0.25 mg/mL trypan blue, 0.9% NaCl, 13 mM citrate buffer pH 4.4) to quench the fluorescence of non-internalized bacteria. After a 1 min. incubation at room temperature fluorescence of the internalized bacteria was quantified on an Victor microplate reader (Perkin-Elmer) with excitation at 485 nm (10-nm band-pass filter) and emission at 530-nm (30-nm band-pass filter). Data were expressed as the percentage of fluorescence of input bacteria that is resistant to trypan blue quenching (fluorescence input bacteria/fluorescence internalized bacteria ×100). Alternatively, bacterial internalization in the presence of inhibitors is expressed as the percentage of internalization of the untreated sample.

### Phagocytosis assay by immunofluorescence microscopy

Phagocytosis assay by immunofluorescence microscopy were performed as by Hilbi *et al.* [46]. An IPTG-inducible GFP-expressing plasmid for *L. pneumophila* was constructed by cloning a EcoRI/HindIII-digested PCR product of gfp+ in pMMB207C giving pXDC31. Gfp-expressing JR32/pXDC31 strain used for the infections was grown to stationary phase in AYE broth containing 0.5 mM IPTG to induce Gfp expression. Before infection, the bacterial cultures were filtered through a 5 µm filter to remove filamentous bacteria and then diluted in RPMI 1640. J774 cells on polylysine-coated round coverslips in 24-well plates were infected with bacteria at an MOI of 20. Alternatively J774 cells were preincubated with polyclonal anti-Legionella antibodies (dilution 1∶200) in complete RPMI 1640 for 30 minutes. The bacteria were centrifuged onto the phagocytes (700 g, 10 min) to synchronize infection and incubated for another 30 min at 37°C. Infected macrophages were washed five times with DPBS and fixed for 15 min with DPBS containing 3.7% formaldehyde hydrolyzed from paraformaldehyde. The fixed cells were washed three times, blocked for 30 min with 5% non-fat milk in DPBS and incubated for 1 h with a rhodamine-conjugated rabbit anti-*L. pneumophila* Philadelphia-1 antibody, diluted 1∶100 in blocking buffer. After washing five times, the coverslips were mounted onto microscopy slides using the mounting media Vectashield Hardset (Vector Laboratories). The samples were viewed with a 100X oil immersion phase-contrast objective on an inverted fluorescence microscope (Nikon Eclipse TE200) equipped with fluorescein and Texas red filter sets.

### siRNA knockdown of CD45 in THP-1 cells

THP-1 cells in exponential growth were electroporated with siRNA duplexes to human CD45 (*ptprc*) obtained from Santa-Cruz biotechnology and Qiagen using Amaxa's Nucleofector® technology. THP-1 cells (1×10^6^ cells) were resuspended in 100 µL of Nucleofector® solution V containing 2 µg of siRNA and electroporated with program V-01. Fresh media containing 10 ng/mL phorbol 12-myristate 13-acetate (PMA) was added to the electroporation cuvette and cells were allowed to recover and differentiate in 96-well (200 µL/well) for 48H. Translocation assays were then performed as described for J774 cells. CD45 protein levels were analyzed by western-blot using anti-CD45 polyclonal antibody (Santa Cruz Biotechnology).

### Differentiation of bone-marrow derived macrophages (BMM)

BMM were obtained from wild-type and *PtprjTM−/TM− Ptprc−/−* knockout C57BL/6 mice. BMMs were prepared by culturing mouse BM cells in BMM media (RPMI-1640 with 10% FCS and 10% culture supernatant from L929 CMG cells producing M-CSF). Macrophages were used for experiments between days 5 and 8 of culture. For translocation and uptake assays, BMMs at day 5 or 6 were plated in 96-well tissue culture plates (Costar) in BMDM media at 1×10^5^ per well. BMM were obtained from two individual mice per genotype.

### Binding assays

Binding assays were performed with BMM plated at 1×10^6^ cells/well in 24-well plates. Cell monolayers were pre-cooled to 4°C and infected with *L. pneumophila* JR32 at an MOI of 20. After centrifugation (1000 g, 10 min., 4°C), *L. pneumophila* was allowed to interact with macrophages for 30 min. at 4°C to prevent phagocytosis. Unbound *L. pneumophila* were removed by five washes with ice-cold PBS, the cell monolayer was then lysed with 1 mL of distilled water and serial dilutions were spread on CYE plates for enumeration of bound *L. pneumophila*.

### Real-time translocation assay in J774 cells

24H prior to infection J774 cells grown in RPMI 1640 (Invitrogen) containing 10% FCS were seeded in black clear-bottom 96 well plate (Corning) at 1×10^5^ cells/well. The day of the experiment J774 cells were pre-loaded with CCF4 as follow. Cells were washed once with CO_2_-independent media (Gibco Invitrogen Corporation, Cat. No. 18045) supplemented with 20 mM L-glutamine. Media then was replaced with 100 µL of CO_2_-independent media supplemented with L-glutamine and 10% FCS (complete CO2IM) plus 20 µL of 6X CCF4/AM solution (6 µM CCF4/AM, 20 mM probenecid in 5% v/v solution B and 67% v/v solution C prepared as described by the manufacturer, LiveBLAzer™-FRET B/G Loading Kit - Invitrogen) and cells were incubated 40 min. at 28°C. Solution B and C are from LiveBLAzer™-FRET B/G Loading Kit and are respectively 100 mg/mL Pluronic®-F127, 0.1% acetic acid in DMSO and 24% w/w PEG 400, 18% TR40 in volume in water. Cells were then washed once with wash media (2 µM CCF4/AM - without solution B and C, 4 mM probenecid, 20 mM L-glutamine in CO_2_-independent media) and wash media was replaced with 120 µL of complete CO2IM containing 2 µM CCF4/AM, 15% v/v solution C and 4 mM probenecid. Bacteria from CYE plates (5 µg/mL chloramphenicol and 0.5 mM IPTG) were resuspended in CO2IM to obtain various MOI (assuming OD = 1 is about 1.4×10^9^ cfu/mL) and each well is infected with 10 µL of the suspension. After centrifugation (900 g, 10 min.) to initiate bacterial-cell contact the plate was plated into a plate reader (Infinite M200, Tecan) preset at 37°C and the infections took place in the plate reader. Cells were excited at 405 nm and fluorescence of substrate (CCF4) and product (released coumarin moiety of CCF4) was recorded respectively at 530 nm and 460 nm at 120 seconds intervals. Assays were carried out in triplicate wells and data were collected with Magellan software v6.4 (Tecan) and then automatically processed in Excel (Microsoft) essentially as described by Mills *et al.* [57] : Data from triplicate wells were averaged and fluorescence values of blank (well without cells) were subtracted from fluorescence values recorded at 460 nm and 530 nm. The 460/530 nm ratio was calculated and smoothed with a moving average in a 5 points window. The product concentration ([P], arbitrary units) were calculated as previously described [57] : [P] = (P_raw_−P_blk_)/(S_0_−S_blk_) where P_raw_ is the product fluorescence measured at 460 nm; P_blk_, background fluorescence at 460 nm; S_0_, measured substrate fluorescence at 530 nm at T = 0; Sblk, background fluorescence at 530 nm. S_0_ normalizes the well-to-well variation in number of J774 cells. The rate of product formation, AppV, values were extracted from the data by subtracting each [P](t) with its predecessor, [P](t-1), divided by 120 seconds, which is the time interval between each two measurements. AppVmax is the highest rate of product formation reached during the translocation process.

## Supporting Information

Figure S1 Effect of CD45 knock-down by siRNA on effector translocation in THP-1 cells. A. Western-blot analysis of CD45 expression level in THP-1 cells after siRNA treatment. B. Translocation of the LepA effector in siRNA-treated THP-1 cells.
(0.29 MB TIF)

Table S1 Complete list of all tested bioactive molecules (Biomol, NINDS and Prestwick collections).
(0.31 MB XLS)

Table S2 List of the 86 molecules from the initial screening which showed more than 50% of inhibition.
(0.04 MB XLS)
