# Supplementary figures and images for: Chemical Genetics Reveals Bacterial and Host Cell Functions Critical for Type IV Effector Translocation by Legionella pneumophila
Source: PLoS Pathog. 2009 Jul 3;5(7):e1000501. doi: 10.1371/journal.ppat.1000501 (PMC2698123; doi:10.1371/journal.ppat.1000501)

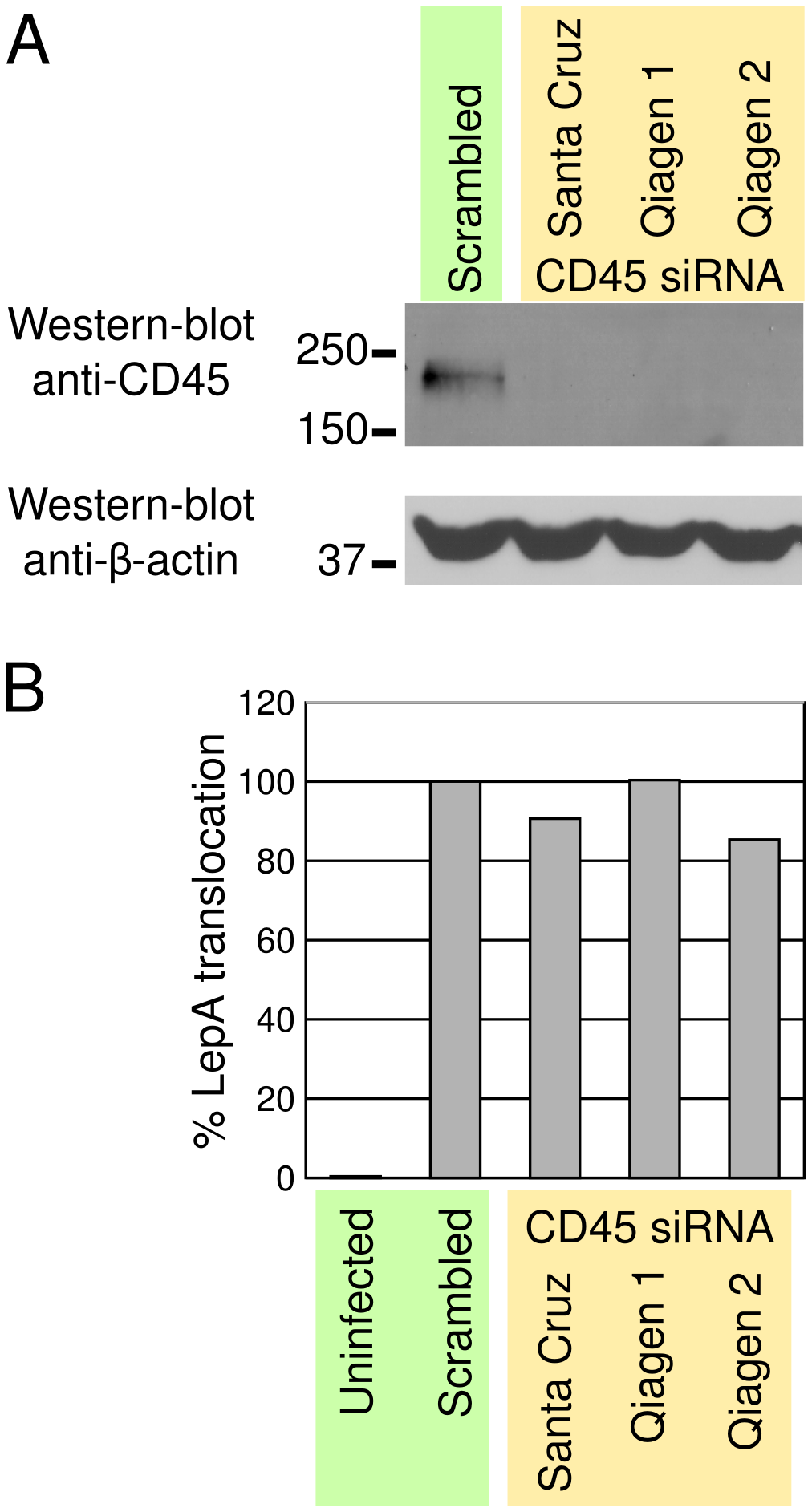

Supplement: Figure S1 — Effect of CD45 knock-down by siRNA on effector translocation in THP-1 cells. A. Western-blot analysis of CD45 expression level in THP-1 cells after siRNA treatment. B. Translocation of the LepA effector in siRNA-treated THP-1 cells. (0.29 MB TIF) [file ppat.1000501.s001.tif]
